# Characterization of the Mechanical Behavior of a Lead Alloy, from Quasi-Static to Dynamic Loading for a Wide Range of Temperatures

**DOI:** 10.3390/ma13102357

**Published:** 2020-05-20

**Authors:** Yann Coget, Yaël Demarty, Alexis Rusinek

**Affiliations:** 1 French-German research institute of Saint-Louis, 5 rue du Général Cassagnou, 68300 Saint-Louis, France; yann.coget@isl.eu; 2 Laboratory of Microstructure Studies and Mechanics of Materials, UMR-CNRS 7239, Lorraine University, 7 rue Félix Savart, BP 15082, 57073 Metz, France

**Keywords:** lead alloy, dynamic mechanical behavior, split Hopkinson pressure bar, ballistic application, dynamic recrystallization, dynamic restoration

## Abstract

The current needs in terms of ballistic protection for armed forces require an almost constant improvement in performance to face the constantly evolving threats and scenarios. Ballistic tests are conventionally carried out in order to assess and validate the levels of protection. The challenge is to be able to set up a digital protocol and only carry out final validation tests. Indeed, the advantage of digital simulation lies in the possibility of being able to evaluate a wide variety of configurations. In order to obtain reliable results, it is necessary to use sufficiently precise material behavior models to transcribe the phenomena observed during the impact. Our study focuses on the behavior of a small caliber ammunition with a ductile core impacting personal protection. More particularly on the mechanical behavior of the lead alloy core. Thus, compression tests have been carried out on a wide range of deformation rates, from quasi-static behavior to dynamic regime, at different temperatures. The study in dynamic conditions was carried out using split Hopkinson pressure bars. Due to the material properties, the experimental device had to be adapted in order to optimize the propagation of the waves allowing to measure signals (elastic waves). These tests demonstrate the dependency of the stress with strain rate and temperature. Dynamic restoration and recrystallization phenomena, characteristic of a material deformed in its hot working area, have also been identified. The associated oscillations due to Pochhammer–Chree effect, observable on the stress–strain curves, constitute the major problem for the implementation of behavioral models. Finally, a constitutive model sensitive to strain rate and temperature is investigated for ballistic purposes.

## 1. Introduction

The constantly evolving threats require improvement in term of ballistic protection, be it to improve performance, decrease weight or make them more comfortable for armed forces. Ballistic tests are conventionally carried out in order to assess and validate the levels of protection. These tests allow to obtain information about the mechanisms occurring during the impact and characterize the behavior of the protection (stopping of the ammunition, perforation, deflection of the rear face, etc.). Nevertheless, it remains a non-optimal and costly solution in time and money (preparation of the different geometries, setting up of tests, limited number of shots, etc.).

The challenge is to be able to implement this protocol numerically and perform only final validation tests. Indeed, the advantage of numerical simulation lies in the possibility of being able to evaluate a wide variety of configurations (materials, geometry, architecture, etc.). The only limitation is the calculation time which can, depending on the complexity of the system, amount to several days. In order to obtain reliable results, it is necessary to use sufficiently precise constitutive models to transcribe the phenomena observed during the impact.

Our study focuses on a small caliber ammunition with a ductile core and more specifically the mechanical behavior of the lead alloy constituting the core of this ammunition. The objective is to set up an experimental protocol from low (0.001–1 s^−1^) to high (700–4000 s^−1^) strain rates and temperature ranging from room temperature to 473 K. Then, the aim is to investigate the experimental results in order to identify a constitutive model suited for ballistic numerical simulation.

The core is the inner part of a jacketed ammunition. The main goal of this part is to provide significant kinetic energy to the ammunition while minimizing the size. Therefore, materials with a high density are mainly used (high mass for a small volume). Lead is known as a heavy metal, its density is higher than most of common materials. It is soft and malleable with relatively low melting point (around 600 K). Thanks to its interesting properties, lead is communally used in ballistics as a filling or core for different types of ammunition. Lead alloys are the best candidates because they are cheap. Usually lead is associated to some additives like antimony, which act as a binder to make the material stiffer [1].

For the study of metal’s plasticity, it is possible to define two areas. On one hand, the cold working zone is characterized by a material temperature being between 0.15 and 0.3 of the melting temperature. On the other hand, the hot working zone can be defined for a material temperature which exceeds half of its melting temperature. In the case of lead, the melting temperature is around 600 K, this is why, at room temperature (around 293 K), the material is in the hot work area. The tests carried out in this study are all above this temperature, the material will be considered as at hot deformations [2]. The hot deformation of metal alloys implies a significant increase of the density of dislocations in each grain. This corresponds to the hardening or the consolidation phenomenon, which results in an increase of the flow stress and the internal energy, and finally induces recovery and recrystallization. Recovery phenomena tend not to alter the grain structure which is elongated by the plastic strain while recrystallization develops new grains by discontinuous nucleation and growth. The occurrence of recovery phenomena, such as dislocation cross-slip, dislocation climb, are referred to as dynamic recovery. For high deformation, this process is generally more pronounced. Recovery is characterized by the rearrangement of the dislocation substructure associated with a partial and gradual reduction of the internally stored elastic energy. This phenomenon leads to a stress–strain curve with a plateau behavior. Dynamic recrystallization describes the formation and motion of new grain boundaries, it mainly occurs during deformation at hot working temperatures. At a certain threshold deformation, nucleation occurs, and new grains are form. Dynamic recrystallization phenomena are characterized by single peak or multiple peak behavior on the stress–strain curve creating a sort of oscillation. The material behavior mainly depends on its stacking defect energy. The examination of the relations between flow stress and equivalent deformation, makes it possible to distinguish two types of metallic alloys: materials with strong or weak energy of stacking defects. In the case of lead alloys, it is subjected to low stacking fault energy [3,4,5]. The alloy additives play also a role on the shape of the stress–strain curve oscillations which can be more or less pronounced.

This study is a challenge in different way. First only few authors studied this material, even more for ballistic purposes. The majority of publications deals with pure lead behavior like Malatynski and Klepaczko [6] who performed a study at high strain rates. They expose the strain rate sensitivity and phenomenon resulting of in instabilities in plastic flow at room temperature. Schmidt and Haessner [7], Hotta et al. [8] were interested by the recovery and recrystallization phenomena. The tests were carried out for different purities of lead and tin alloys. They show the composition influence on the stress–strain curve and on recrystallization behavior. Peroni et al. [9], Wiśniewski and Pacek [10] focused on the simulation of ammunition impact onto different protections systems, using lead-antimony material. They show the leak of results for lead alloys in literature and especially for high strain rate. Globally, the results obtained for this kind of alloys showed significant strain-rate sensitivity in the range covered with the Split Hopkinson Pressure Bar (SHPB) tests. The shapes of the stress–strain curves are very different in quasi-static and dynamic regime.

Moreover, even if the mechanical characterization of metals like steel or aluminum is well mastered [11], the particularly soft mechanical behavior of lead can pose difficulties either for mechanical testing or production of specimens. Indeed, these latter are extracted by machining of the small caliber ammunitions which is a non-straightforward procedure. The maximum size of cylindrical samples which can be obtained is 7 mm × 7 mm for a 9 mm caliber. These limited dimensions make the experimentation more complicated especially for the dynamic testing.

Finally, as the samples are obtained from real ammunitions, the number of specimens is limited which prevent the possibility of large test campaign.

## 2. Experimental Study

### 2.1. Material Properties

**Figure 1:**
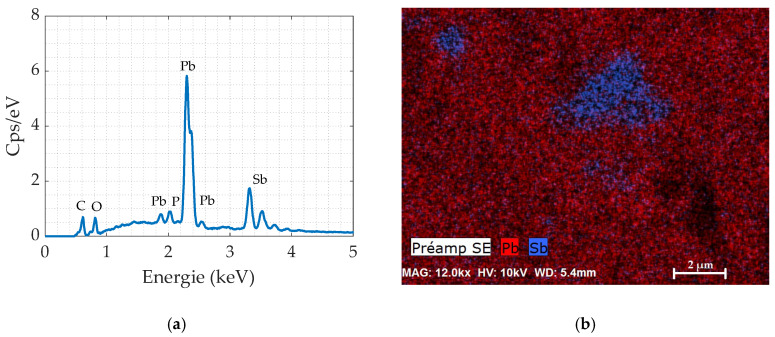
Energy-dispersive X-ray Spectroscopy (EDX) of the lead alloy: (**a**) EDX analysis; (**b**) EDX mapping.

Firstly, in the aim to get knowledge on the composition of the lead alloy core, elemental microanalysis was performed in a Field Emission Scanning Electron Microscope (FE-SEM Thermofisher Scientific NNS450, Hillsboro, OR, USA) with Energy-dispersive X-ray spectrometry (EDS-Bruker SDD XFlash 6130, Karlsruhe, Germany). Figure 1 presents (a) the EDS spectrum with the identification of Pb, Sb, P as main elements and (b) the EDS mapping showing antimony inclusions within a lead matrix.

The hardness of the lead alloy is studied in order to evaluate the homogeneity of the ammunition’s core. Since the material is small and soft, a Vickers indentation appears to be the most relevant method to study the hardness. The tests are carried out using Buehler’s micro hardness tester Wilson VH1202 (Esslingen am Neckar, Germany).

**Figure 2:**
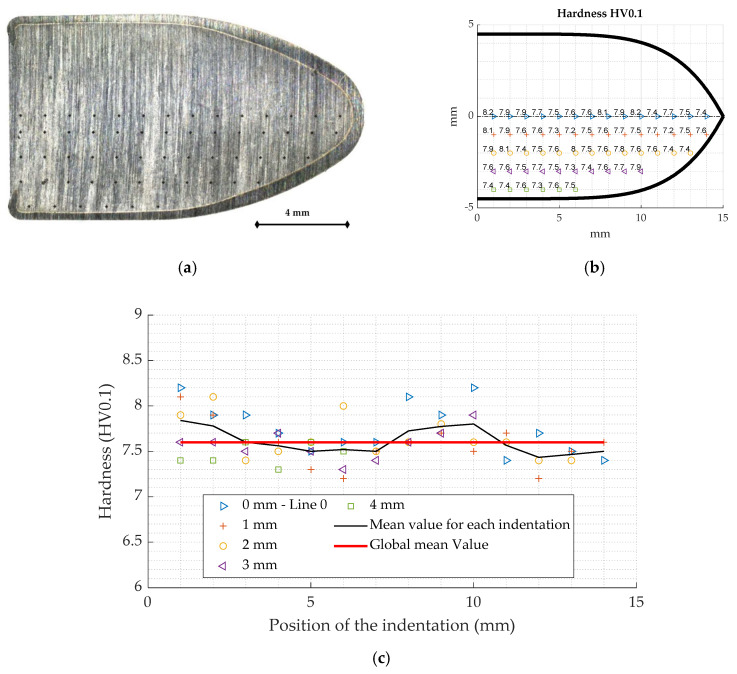
(**a**) Longitudinal section of the 9 mm ammunition with the indentation points; (**b**) hardness mapping in the longitudinal side of the ammunition (HV0.1); (**c**) hardness for the different indentation and average.

To achieve the hardness test on the specimen, a transverse cutting of the ammunition is achieved (Figure 2a). A polishing is carried out in order to obtain good surface condition. An indentation mapping is performed using a 0.1 kg loading with a hold time of 10 s to check the homogeneity along the longitudinal axis of the material and to determine an average hardness value.

The study of the hardness values (Figure 2b,c) shows that the material is homogeneous and has an average hardness of 7.6 HV0.1. In addition, comparative tests carried out on pure lead samples (Goodfellow-LS512679-99.95% pure lead) show mean hardness values of 4 HV0.1, meaning that the inclusion of additives in the core nearly doubles its hardness.

Secondly, the density of the material is measured using Archimedes method which consists in measuring the specimen mass in the air ($m_{air}$) and in a liquid ($m_{liquid}$). Thus, using Equation (1) the density of the material is defined.

(1) $$\rho =\frac{m_{air}}{m_{air}-m_{liquid}}\left(\rho_{liquid}-\rho_{air}\right)+\rho_{air}$$

Finally, the different elastic mechanical parameters are obtained by an ultrasound method: Young’s modulus (*E*), Poisson’s ratio (ν), and Shear Modulus (*G*). This method consists in measuring the propagation speeds of the transverse $\left(V_{T}\right)$ and longitudinal $\left(V_{L}\right)$ waves in the material using an "Olympus 38DL PLUS 45MG" device (Waltham, MA, USA). The Poisson’s ratio (Equation (2)), Young’s modulus (Equation (3)), and shear modulus (Equation (4)) are obtained by the following relations:

(2) $$\nu =\frac{1-2{\left(\frac{V_{T}}{V_{L}}\right)}^{2}}{2-2{\left(\frac{V_{T}}{V_{L}}\right)}^{2}}$$

(3) $$E=\frac{V_{L}^{2}\rho \left(1+\nu \right)\left(1-2\nu \right)}{1-\nu }$$

(4) $$G=V_{T}^{2}\rho$$

The values are reported in Table 1.

As it is exposed in the Table 1, the material has a very high density 10,940 $\mathrm{kg}/m^{3}$ compared to the ones of steel (7800 kg/m^3^) or aluminum (2700 kg/m^3^). The Poisson’s ratio is also high (0.4) as a commonly encountered value for metals is found around 0.3. Finally, the Young’s modulus is relatively low (25.5 GPa) making the material more elastic compared to a typical steel (210 GPa) being 8 times higher. These properties make this material suited for ammunitions giving a high kinetic energy (Equation (5)), minimizing the volume of the projectile and helping the ammunition to be stabilized during the flight period.

(5) $$E_{kinetic}=\frac{1}{2}.\rho .V.v_{i}{}^{2}$$

where *V* is the volume of the ammunition and $v_{i}$ the initial velocity of the projectile.

### 2.2. Mechanical Characterization

#### 2.2.1. Specimen Geometry

**Figure 3:**
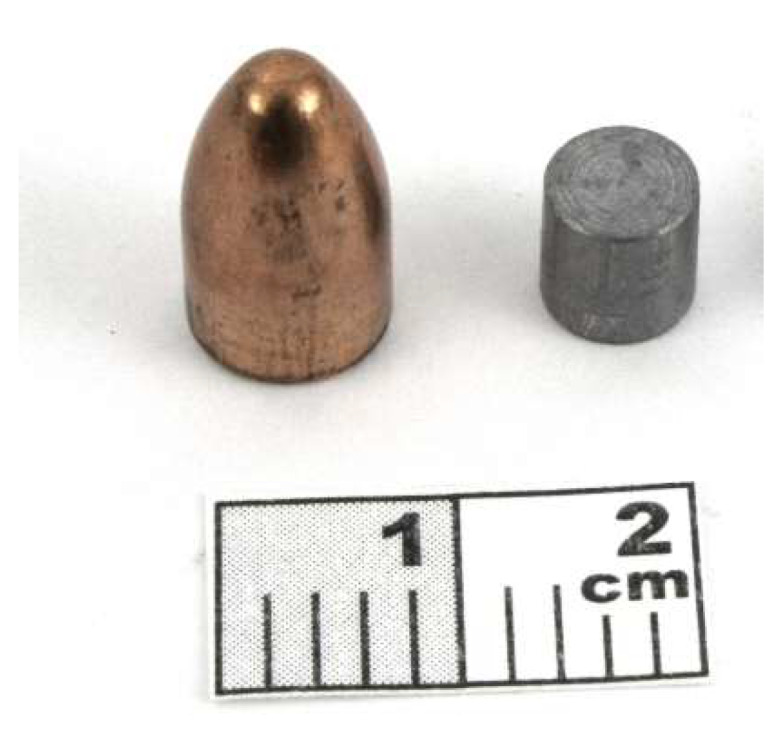
Picture of the specimen extracted from the ammunition.

In the aim to characterize the mechanical properties of the lead core, cylindrical samples are extracted by milling of the bullet. Samples have a size of 7 mm for the diameter and 7 mm in height to have a ratio of 1, see Figure 3. This restriction allows to reduce friction effect and motion [12].

Once the geometry of the samples has been defined, the next step of the study is focused on the experimentation.

#### 2.2.2. Quasi-Static Test under Compression Loading

##### Experimental Protocol

The first step in the characterization process, is to carry out compression tests in quasi-static regime. To do it, the tests are achieved on a universal electromechanical press Instron equipped with 100 kN load cell. A video camera as well as a lightening device are used to record the local strain of the sample during the tests. A “shadowing” method is applied consisting in having a black–white contrast between the sample and the background. These images are used to evaluate if a barrel effect occurs during the test. Lubrification with petroleum jelly is done to prevent this phenomenon and to keep longer the homogeneous process of deformation. As a first step of the tests, a tool-tool compression test without sample is performed so as to remove potential effects of the rigidity of the machine, through a commonly named compliance correction.

In order to characterize the material mechanical behavior over a wide range of conditions, the tests are performed for strain rates going from 0.001–1 s^−1^ and temperatures from 20–200 °C. The experimental conditions are summarized in Table 2 and Table 3, respectively. As the quantity of available material is limited, only test 3 is repeated 3 times to verify repeatability. Then, to be able to study a large range of strain rates and temperatures, only one test per condition is carried out. The temperature tests are performed in a climatic chamber, the initial temperature of the tested specimen being controlled by a thermocouple stocked on them. Finally, the machine is controlled by strain rate, and the measured force-displacement field is recovered to study the material behavior.

##### Experimental Results and Analysis

In order to evaluate the influence of the machine stiffness, compliance tests are carried out. Force and displacement are respectively measured by the load cell and traverse displacement of the machine. The MATLAB “Curve fitting tool” is used with the Equation (6) to determine the relation between force and displacement. The obtained values of the parameters a and n are given in the Table 4. Then the specimen displacement can be corrected using Equation (7) [13].

(6) $$D_{machine}=aF^{n}$$

(7) $$D_{cor}=D_{exp}-D_{machine}$$

where $D_{machine}$ corresponds to the displacement of the traverse of the machine, F the applied force, $D_{exp}$ is the displacement measured when testing a sample and finally $D_{cor}$ is the corrected displacement.

**Figure 4:**
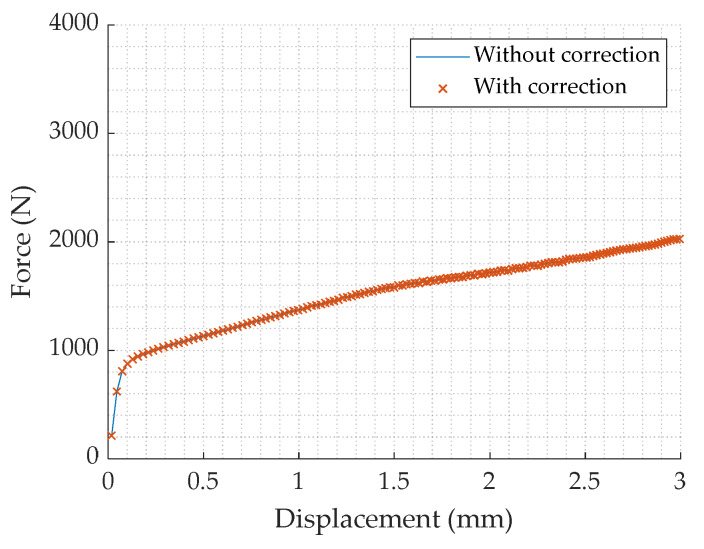
Force–displacement curve of a compression test on a lead alloy sample, at 0.01 s^−1^ room temperature, with and without compliance correction.

The comparison of the force–displacement curves with a lead alloy sample, measured by the load cell, with and without correction (Figure 4) shows that there is few influence of the machine stiffness. This is quite understandable as the studied material is very soft.

**Figure 5:**
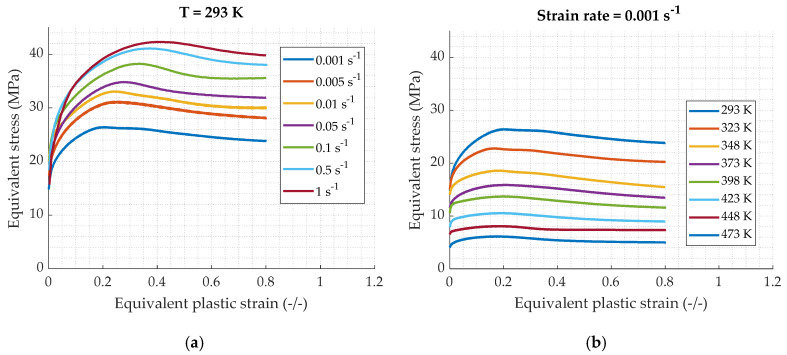
True plastic strain–true stress curves for quasi-static compression loading: (**a**) strain rate influence for a constant temperature of 293 K; (**b**) temperatures influence for a constant strain rate of 0.001 s^−1^.

The true plastic strain-true stress curves at 293 K for different strain rates are exposed in Figure 5. A strong strain rate dependency is observed. In fact, an increase of the strain rate produces an elevation of the stress. Tests carried out at a constant strain rate of 0.001 s^−1^ for different temperatures are shown in Figure 5b. Similarly, the material exhibits a significant sensitivity to the temperature; an increase of temperature producing a decrease of the stress. Moreover, it can be visible that the curves obtained at room temperatures (Figure 5a) meet a maximal peak followed by a decrease of the stress which seems to reach a stationary domain as explained in Section 1. Thus, the results highlight the dynamic recrystallization behavior of the material. In addition, a beginning of characteristic oscillations can be observed for example on the 0.1 s^−1^ curve. For this range of plastic strain, one peak can be observed. At a constant strain rate and for different temperature, the peak abscise stays at a constant value. As the strain rate increases for a constant temperature, the peak occurs for a higher plastic strain.

### 2.3. Dynamic Test under Compression

#### 2.3.1. Design of the Dynamic Setup

**Figure 6:**
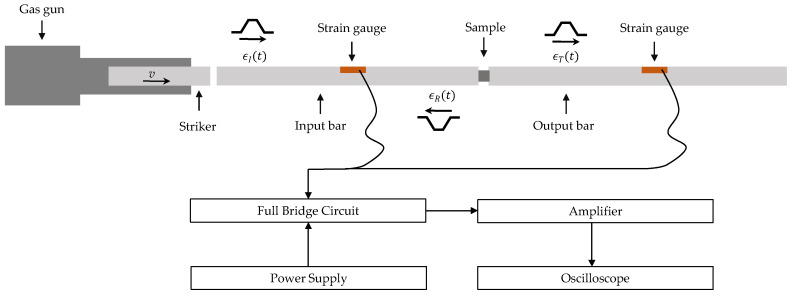
A schematic illustration of split Hopkinson bar pressure (SHPB) apparatus.

The second step of the mechanical characterization consists in performing compression tests in dynamic regime so as to study the strain rate effect on the material behavior. For this purpose, split Hopkinson bar pressure (SHPB) tests may be done [14]. A typical SHPB system is composed of 3 bars: a striker, an input bar, and an output bar (Figure 6) [15]. The striker is impacting the input bar using a compressed air gun. This generates an elastic incident compressive wave ($\epsilon_{i})$ which is propagated along the input bar. Once the incident wave reaches the input bar–specimen interface, the wave is divided into two elastic waves: a reflected wave ($\epsilon_{r}$) in the input bar and a transmitted wave ($\epsilon_{t}$) which propagates through the output bar. A full-bridge of four strain gauges is glued on the input and output bars to measure the different elastic waves.

**Figure 7:**
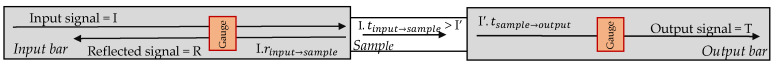
Signal transmission in SHPB setup.

In order to obtain exploitable signals from the gauges, the SHPB setup parameters have to be carefully considered. As far as the sample and bar material properties are concerned, they have an influence on the transmission and reflection of waves, see Figure 7. To study the transmission and reflection coefficients, the impedances have to be determined using Equation (8). The values obtained for different materials are summarized in Table 5. Most commonly used materials in SHPB setups have been reported in this table. As we can see, lead and aluminum have similar impedances, as the steel value is the double, nylon having the smaller impedance which is ten times lower than lead.

(8) $$Z=\sqrt{\rho \times E}$$

where *Z* is the material acoustic impedance in kgm^−2^s^−1^.

The transmission and reflection coefficients are calculated from material impedances, using Equations (9) and (10). The results are displayed for different interfaces between: input bar–sample (Table 6) and sample–output bar (Table 7).

(9) $$t_{1\to 2}=\frac{2\times Z1}{Z1+Z2}$$

(10) $$r_{1\to 2}=\frac{Z1-Z2}{Z1+Z2}$$

where $t_{1\to 2}$ is the transmission coefficient for a wave going from solid 1 to solid 2 at the interface 1/2 and $r_{1\to 2}$ is the reflection coefficient for a wave reflecting at the 1/2 interface. Z1 and Z2 are respectively the impedance of materials 1 and 2.

For the input bar–sample interface, it is necessary to have a sufficient transmission to get a measurable magnitude of the output signal as well as a good reflection which will provide the required reflected signal. The best compromise seems to be an input bar made of steel since it has the highest values (t = 1.448, r = 0.448) compared with the other materials.

For the sample–output interface, only the transmission wave is needed. The best coefficient is obtained for the nylon bar. Nevertheless, it is more complex to post process signals for visco-elastic bar setup [16] and even more when there is a combination metal/nylon. Other options, as tubes could have been investigated, but the goal is to put in place the simplest setup. This is why these options are not considered for the present study. An alternative could be found with the aluminum which has a transmission coefficient of 1.075.

Secondly, as preliminary solutions have been found, numerical simulations are done with different types of bars (Table 8). The goal is to determine which configuration could offer the best signals. In fact, two setups are available in our laboratory with a diameter of 20 mm and 12 mm. Thus, it is possible to play with the bar diameter and material.

To start with a simple model, a perfect plastic behavior of the lead alloy (see Equation (11)) is supposed based on the quasi-static results. As they show that the stress increases with the strain rate, it is possible to suppose a yield stress (value $\sigma_{y}$) of about 50 MPa.

(11) $$\;\sigma <\sigma_{y}\;:\;\sigma =E.\epsilon \;\sigma >\sigma_{y}\;:\;\sigma =\sigma_{y}$$

**Figure 8:**
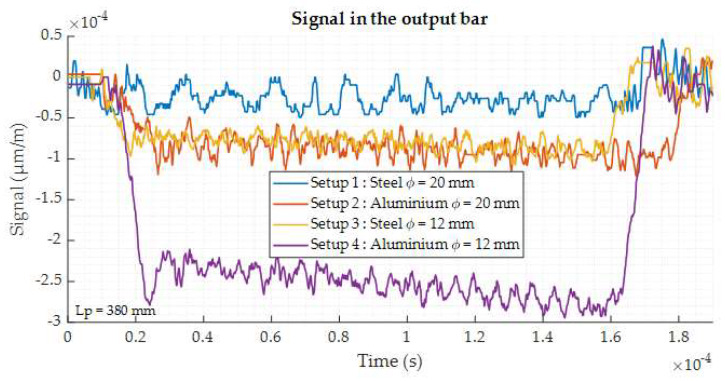
Output signal for the different setups obtained by numerical simulation.

The numerical simulation is performed in Abaqus^®^ Explicit using an axisymmetric modeling with a mesh size of 2.53 mm^2^ respecting the aspect ratio of 5. The initial striker velocity is fixed at 10 m/s which is the minimum speed which can be applied with our available setups. This is considered as the most unfavorable case as it leads to the lowest amplitude of the gage signals. The cylindrical sample have the same geometry as for the quasi-static tests: 7 mm in diameter and 7 mm in height. Numerical gauges are created at the same location as in the experimental setups, on the input and output bars. The related numerical signals recorded on the output bar are displayed on the Figure 8.

**Figure 9:**
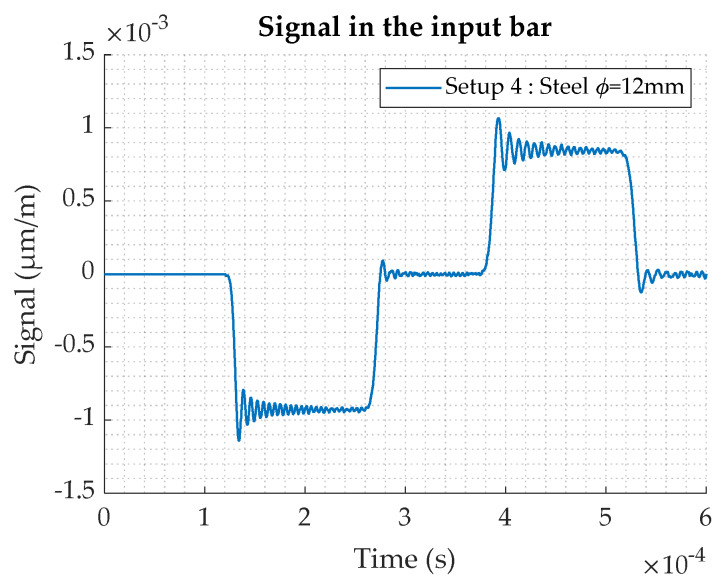
Input signal for the Setup 4, obtained by numerical simulation.

The results of the simulation show the influence of the output bar geometry. Indeed, as the diameter decreases, the signal becomes higher. For the Setup 1 (ϕ = 20 mm—steel–steel), the signal is very low. The Setup 3 (ϕ = 12 mm—steel–steel) starts to give a more significant signal. If aluminum is considered for the output bar material, the signal amplitude is increased. The output signal of the Setup 2 (ϕ = 20 mm—steel–aluminum) is similar to the one obtained with the Setup 3 (ϕ = 20 mm—steel–steel). Finally, the highest amplitude is reached with the Setup 4 (ϕ = 12 mm—aluminum). The value is increased by a factor 2.5 compared to Setups 2 or 3. The Figure 9 shows the input signal for the Setup 4. It appears from the numerical study that the Setup 4 is the best solution, as both the input and output signals are significantly improved in comparison to the other configurations.

**Figure 10:**
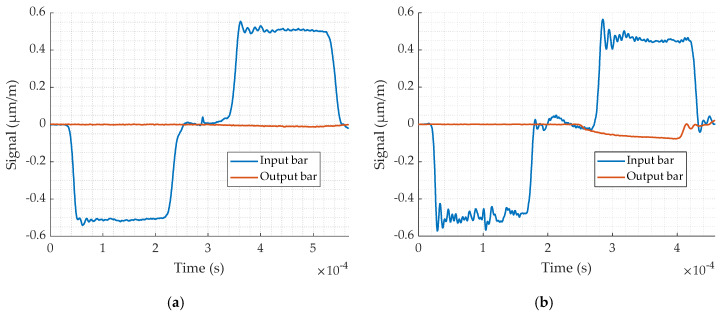
Input and output signals obtained by experimental testing for: (**a**) Setup 1; (**b**) Setup 4.

Finally, two experimental validation tests are carried out, one on the available Setup 1 and the other on Setup 4 (see Table 8). The comparison between the two tests (Figure 10 confirms that Setup 4 gives the best result as it was predicted by the numerical test. In conclusion, Setup 4 has been selected for the present study of the lead alloy.

#### 2.3.2. Experimental Protocol

**Figure 11:**
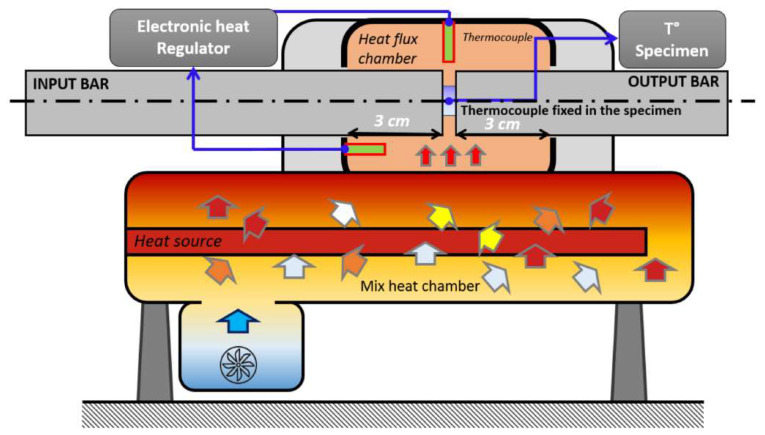
Schema of the oven used for the test using SHPB at high temperatures [11].

The dynamic tests are performed for strain rates from 1200–3800 s^−1^ and temperatures from 20–200 °C. The related experimental conditions are summarized in Table 9 and Table 10. The temperature tests are made in a furnace which has been specifically designed for SHPB tests. The scheme representing its operating mode is given in Figure 11 [11,12,13,14,15,16,17]. The extremities of the two bars are initially heated to the desired temperature during 30 min. A thermocouple glued on a sample gives the initial temperature of the outer surface of sample. To maintain 200 °C (the highest tested temperature) inside the specimen, 5 min of waiting time were needed. As the other tested temperatures were lower, the specimen has been heated during this same amount of time.

#### 2.3.3. Experimental Results and Analysis

The one-dimensional elastic wave propagation theory based on uniform deformation is supposed to determine the strain in the specimen (Equations (12)–(15)). First, the strain rate can be computed with Equation (12).

(12) $${\dot{\epsilon }}_{S}=\frac{C_{input}*\left(\epsilon_{r}-\epsilon_{i}\right)-C_{output}*\epsilon_{t}}{L_{s}}$$

where $C_{input}$ and $C_{output}$ are the wave speeds in the input and output bars, $L_{s}$ is the sample length.

Strain in the specimen is determined by integrating the strain rate (Equation (13)).

(13) $$\epsilon_{s}=\overset{t_{f}}{\underset{0}{\int }}{\dot{\epsilon }}_{sample}dt$$

Assuming force equilibrium leads to:

(14) $$\epsilon_{reflected}=\epsilon_{transmitted}-\epsilon_{incident}$$

(15) $$\sigma_{s}=\frac{E_{output}*S_{output}*\epsilon_{transmitted}-E_{input}*S_{input}*\left(\epsilon_{incident}+\epsilon_{reflected}\right)}{2*S_{sample}}\;\sigma_{s}=\frac{(E_{output}*S_{output}+E_{input}*S_{input})*\epsilon_{transmitted}}{2*S_{sample}}$$

where, *S* corresponds to the different sections.

**Figure 12:**
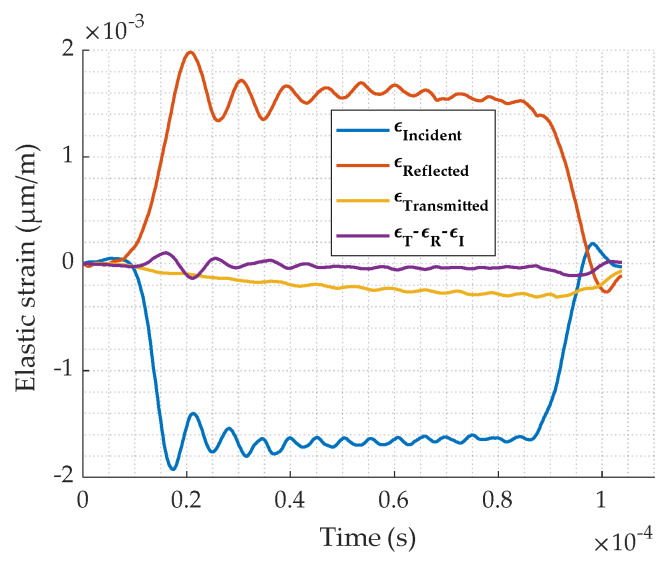
Waves equilibrium verification for test at 1850 s^−1^.

In order to check the force equilibrium hypothesis in our experiments, the incident, reflected, transmitted strain are plotted in Figure 12. Equation (14) is also plotted and shows that its value is near zero which means that the equilibrium is fulfilled. As a result, the strain–stress can be calculated for this study.

Finally, considering that the input and output bars have the same diameters, the following simplified equation is obtained and will be used to obtain the stress in the sample.

(16) $$\sigma_{s}=\frac{(E_{output}+E_{input})*S_{bar}*\epsilon_{transmitted}}{2*S_{sample}}$$

**Figure 13:**
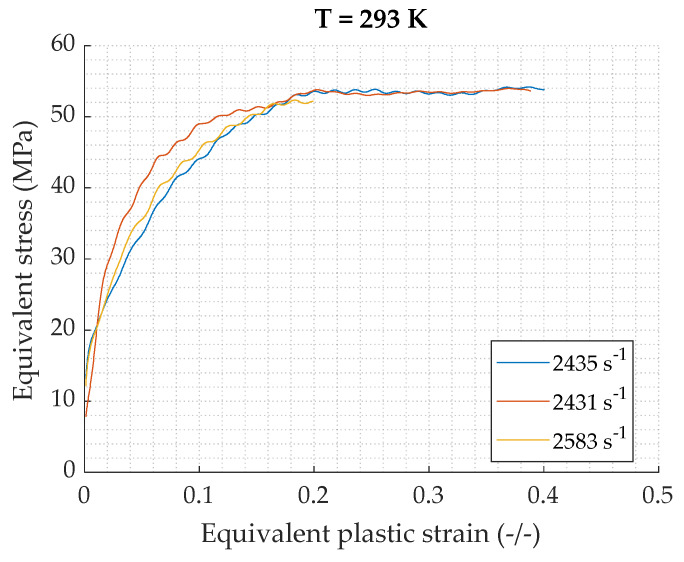
Three identical samples tested in dynamic compression with the same striker velocity.

To evaluate the repeatability of the tests, 3 tests are carried out at approximately 2500 s^−1^. Results are shown on the Figure 13 and are pretty similar, especially for the stationary domain.

The influence of inertia and friction [18] is evaluated in this study. The stress caused by these two phenomena can be calculated using Equations (17) and (18).

(17) $$\sigma_{friction}=\frac{\mu l\sigma }{3d}$$

(18) $$\sigma_{inertia}=\frac{\rho d^{2}}{12}\left({\left(\frac{d}{l}\right)}^{2}-\frac{3}{12}\right)\left({\ddot{\epsilon }}^{2}+\dot{\epsilon }\right)+\frac{3\rho d^{2}}{64}\ddot{\epsilon }$$

(19) $$\sigma_{corrected}=\sigma -\sigma_{inertia}-\sigma_{friction}$$

where *l* and *d* are the specimen length and diameter, μ is the friction Coulomb coefficient (< 0.02), and $\ddot{\epsilon }$ the acceleration.

**Figure 14:**
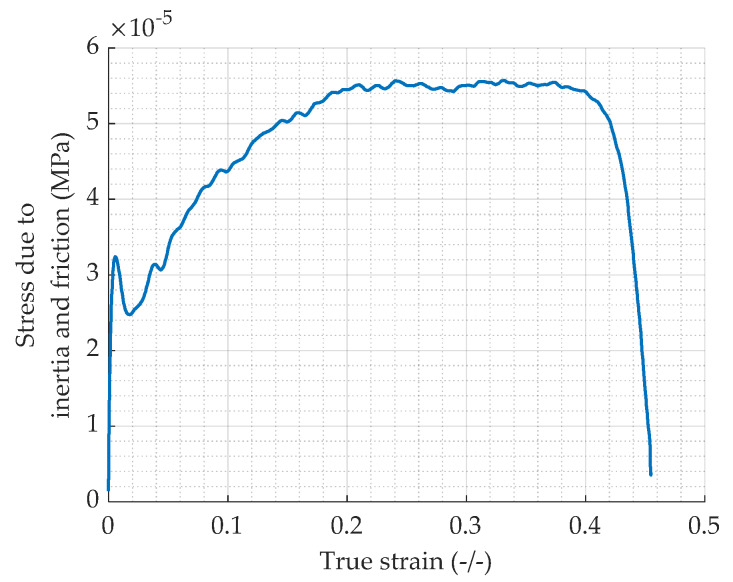
True stress induced by inertia and friction as defined in Equation (23).

The stress due to inertia and friction in function of the true strain is plotted for the highest achieved true strain rate. Figure 14 shows that the values are very low, about 10^−5^ MPa. This is consistent as the dimensions of the specimen are very small (7 × 10^−3^ m in diameter and height) and as in Equation (18), the diameter is at the power two which makes the influence of inertia and friction even lower. As a conclusion, the inertia-friction correction (Equation (19)) is not justified and will not be considered for the case of the lead SHPB tests as confirmed by [6].

**Figure 15:**
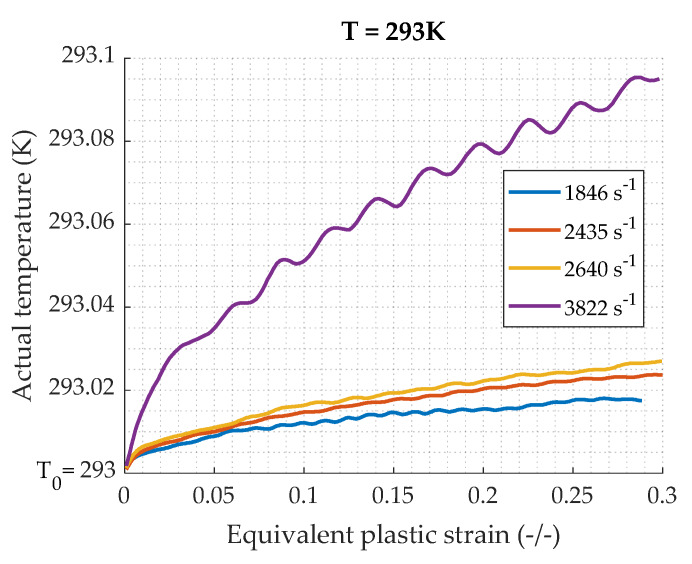
Temperature variation in the specimen during the test at constant temperature.

As far as dynamic testing is concerned, a part of the plastic work is converted into heat and cannot be dissipated along the specimen, corresponding to adiabatic heating. The temperature increase during the process of plastic deformation can be calculated by Equation (20). Figure 15 shows the calculated temperature rise of the specimen during each test. This indicates that the temperature increasing during tests is very small, the maximal difference being 0.1 K. This small increase is logical as the stress values are low and the density is quite important. The temperature effect correction is not necessary for the study of this lead alloy.

(20) $$\Delta T=T-T_{0}=\frac{\beta }{C_{p}.\rho_{s}}\int_{\epsilon_{e}}^{\epsilon_{p}}\sigma d\epsilon$$

where $C_{p}$ is the specific heat at constant pressure, $\rho_{s}$ the specimen density, $T_{0}$ and T are respectively the initial and the temperature during the test of the specimen. β is the Taylor–Quinney coefficient which corresponds to the part of the plastic work converted into heat. For metals, it is usually equal to 0.9 (Table 11) but this value is open to controversy; other methods exist to determine this coefficient [19]. However, for the lead alloy, this value is not critical as the density of the material is very high.

**Figure 16:**
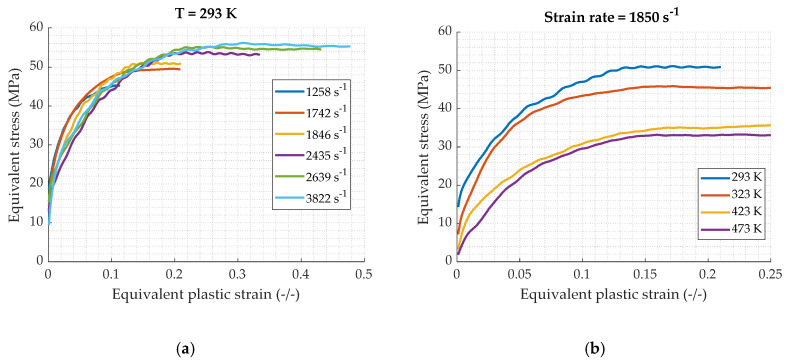
True plastic strain–true stress curves in dynamic compression loading: (**a**) at different strain rates and constant temperature of 293 K; (**b**) at different temperatures and constant strain rate of 1850 s^−1^.

The final true stress–true plastic strain results are displayed on Figure 16. The same trends as in quasi static regime can be observed. In fact, the stress increases with the strain rate and decreases with the elevation of temperature. Furthermore, the strain rate sensitivity is less pronounced at higher strain rate. As can be seen between 2640 s^−1^ and 3822 s^−1^, there is an increase of 2 MPa. There is 15 MPa between 0.001 s^−1^ and 1 s^−1^ (Figure 5). Contrary to the lower strain rates, the oscillations on the strain–stress curves are no longer observable and the stress reaches immediately a plateau. This is the result of the restoration behavior which occurs at higher strain rates (as explained in Section 1).

### 2.4. Analysis of the Temperature and Strain Rate Dependencies

**Figure 17:**
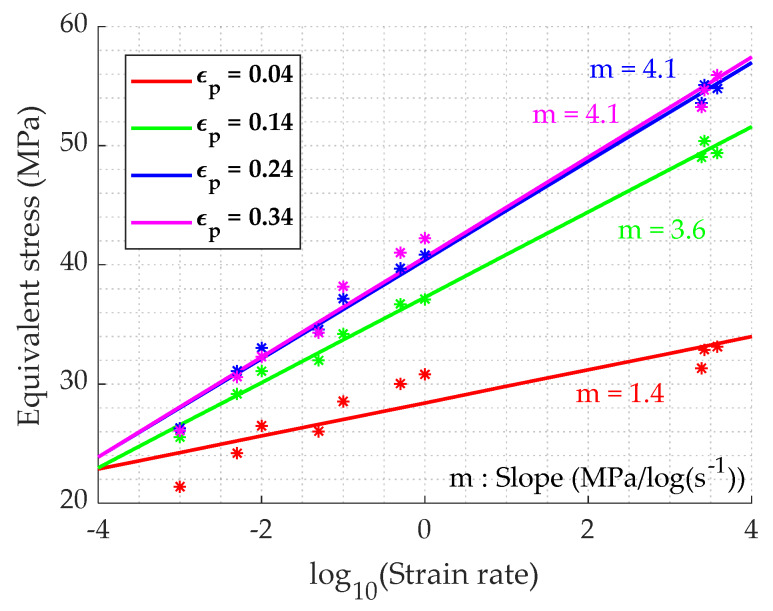
True stress in function of strain rates, at room temperature, for different values of the true plastic strain.

**Figure 18:**
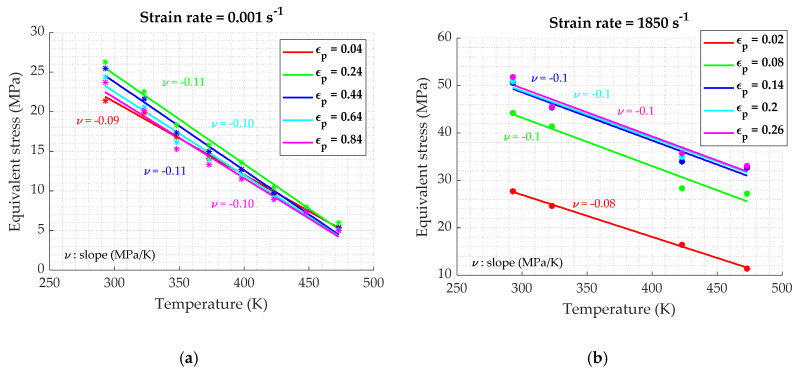
Temperature–stress for different strains: (**a**) in quasi-static conditions; (**b**) in dynamic conditions.

Once the different experimentations are completed, relations between stress, strain, temperature and strain rate have to be investigated in order to understand the material behavior [20]. Figure 17 points out the stress in function of the logarithmic strain rate, at different values of plastic strain. For a constant value of strain, the stress seems to increase linearly. The slope is dependent on the strain, its value increasing and stabilizing as the strain increases, reaching the stationary domain of the stress–strain curve. As it is exposed on Figure 18, the temperature sensitivity differs at low and high strain rates. In fact, for the low strain rates (Figure 18a), the stress–temperature relation could be approximated by a linear function, with a constant slope with the strain. While in dynamic conditions (Figure 18b), it seems to follow an Arrhenius equation of the form ($\sigma =A\cdot e^{\frac{-k}{T}}$) with a dependency to strain levels.

The different tests carried out on the lead alloy at quasi-static and dynamic strain rates for different temperatures, allowed to understand the sensitivities of the material. Thanks to this, the next section deals with the implementation of a constitutive model for ballistic needs.

## 3. Constitutive model

### 3.1. Objective

The material mechanical properties have been studied in order to determine its behavior at different strain rates and temperatures. The next step is to adapt a constitutive model which can be used to simulate ballistic impacts. For this purpose, a model based on the theory of dislocations would be particularly appropriate, as it was proposed by Zerilli Armstrong [21], Goto et al. with the Mechanical Threshold Stress (MTS) model [22], Rusinek and Klepaczko [23], or more recently by Langer, Bouchbinder, Lookman [24] and developed by Le, Le, Tran [25]. However, in this paper, only empirical constitutive models are considered as a first approach based on the original and complex experiments performed using lead material.

### 3.2. Perfect plastic modified model for ballistic application

As it was exposed in the previous results, the temperature and strain rate dependencies do not allow to use the Johnson–Cook model (Equation (21)) [26], which is one of the most popular for metallic materials in such applications. In fact, this model is linear in term of strain rate sensitivity. Moreover, it is a strictly increasing monotonic function which does not allow to reach the restoration plateau.

(21) $$\sigma =\left[A+B\varepsilon^{n}\right]\left[1+C.ln\left(\frac{\dot{\varepsilon \;}}{\dot{\varepsilon_{0}\;}}\right)\right]\left[1-{\left(\frac{T-T_{0}}{T_{f}-T_{0}}\right)}^{m}\right]$$

During the impact, the bullet is subjected to high velocities, inducing high strain rates in the material, and also large deformation as we can see on the tests carried out in [27]. Therefore, for the lead core a perfect plastic behavior is assumed and defined by Equation (22). As can be seen on the dynamic test results (Figure 16), the stress reaches a stationary value which is defined as the yield stress in the present model. Figure 17 shows that the stress is linear to the logarithmic strain rate, which means that the stress can be described by a power low of the strain rate. For the temperature dependency, at high strain rate, an Arrhenius formulation form is chosen in accordance with the trend observed in Figure 18b.

(22) $$\sigma <\sigma_{y}\;:\;\sigma =E.\epsilon \;\sigma >\sigma_{y}\;:\;\sigma =\sigma_{y}$$

where the yield stress $\sigma_{y}$ is define as follows, Equation (23).

(23) $$\sigma_{y}=A.{\left(\frac{\dot{\varepsilon \;}}{\dot{\varepsilon_{0}\;}}\right)}^{n}.\exp\left(-\frac{k}{T}\right)$$

where, *A* is a constant stress, $\dot{\varepsilon \;}$ is the strain rate, *k* is a temperature parameter, and *T* is the current temperature. The values obtained for the lead alloy are given in the Table 12. 

**Figure 19:**
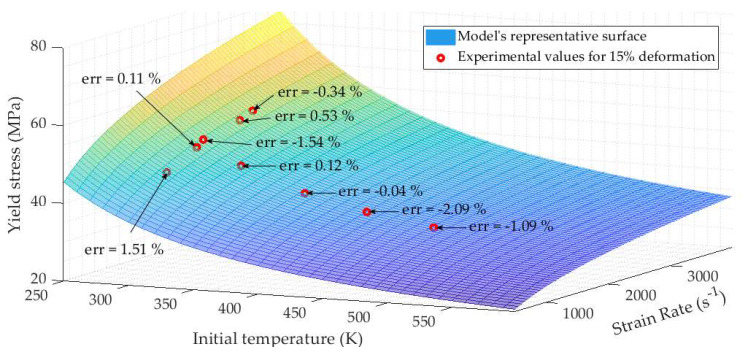
3d representation of the yield stress field and experimental points.

Figure 19 is a 3d representation of the yield stress model. The highest stress is obtained for the lowest temperature and highest strain rate. The lowest values of the stress are achieved at the fusion temperature and the lowest strain rate which is consistent with the mechanical behavior of a classical material. Experiment points defined at 15% of deformation representing the beginning of the plateau behavior are plotted on this figure in order to compare the model with real data. The correlation is satisfactory as the differences between experimental and analytical values are very low; the mean error percentage being close to 0.8%.

## 4. Conclusions

To conclude, this study proposes an experimental protocol to study the mechanical behavior of a soft metallic material, being a lead alloy. It also presents a simple constitutive model of a lead alloy which could be used for ballistic purposes. Classical quasi static tests were carried out on small cylinder 7 × 7 mm^2^ directly extracted from the bullets. Dynamic tests were done using a SHPB facility with a setup which was specifically designed for this study. It was made of bars of diameter 12 mm, the striker and input one in steel, the output bar was in aluminum which allowed to optimize the measured amplitude of the gage signals. This adapted setup is made of materials which are commonly used for SHPB tests and do not require a complicated post treatment calculation to convert signals to stress-strain curves.

The tests carried out at different temperatures and strain rates exposed a significant sensitivity to these parameters. Moreover, the stress–strain curves presented a different shape at low (small oscillations) and high strain rates (plateau) which are characteristic of recrystallization and dynamic recovery behavior.

Finally, considering the material temperature and strain rate dependencies, the Johnson–Cook constitutive model was determined as not suited for this study. That is why a perfect plastic constitutive model, sensitive to strain rate and temperature, was preferred. In fact, it was assumed that the material solicitations are mainly at high deformations and high strain rates during a ballistic impact. This model showed a good correlation with the experimental results.

In the aim to simulate the impact of a bullet, composed of a lead alloy core, on a protection system, the mechanical behavior of the lead alloy under shear and tension solicitations should be considered. Damage and failure criteria would also be necessary. Thus, shear and traction tests would be interesting. Nevertheless, as for this study, it could be a challenge because there are few results in the literature and the size of the specimen make the experimentation complicated.

## Figures and Tables

**Table 1:** Lead alloy core’s material parameters.

Density (kg/m^3^)	Poisson’s Ratio	Young’s Modulus (GPa)	Shear Modulus (GPa)
10,940	0.40	25.5	9.1

**Table 2:** Quasi-static test conditions at room temperature (293 K).

T = 293 K	Test 1	Test 2	Test 3	Test 4	Test 5	Test 6	Test 7
Strain Rate (s^−1^)	0.001	0.005	0.01	0.05	0.1	0.5	1

**Table 3:** Quasi-static test conditions for a constant strain rate (0.001 s^−1^).

$\dot{\epsilon \;}=0.001\;s^{-1}$	Test 1	Test 2	Test 3	Test 4	Test 5	Test 6	Test 7	Test 8
Temperature (K)	293	323	348	373	398	423	448	473

**Table 4:** Values of the compliance parameters.

a (mm/N)	n
3 × 10^−4^	0.654

**Table 5:** Impedances for different materials.

Material	Lead	Steel	Aluminum	Nylon
Young modulus (GPa)	25.5	234	73	2.9
Density ($\mathrm{kg}/m^{3}$)	10,940	8075	2830	1150
Impedance $\left(10^{6}{\mathrm{kgm}}^{-2}s^{-1}\right)$	16.7	43.4	14.3	1.82

**Table 6:** Transmission and reflection coefficients between input bar and lead specimen.

Interface	Steel → Lead	Aluminum → Lead	Nylon → Lead
Transmission coefficient	1.448	0.9250	0.1971
Reflection coefficient	0.448	−0.075	−0.8029

**Table 7:** Transmission and reflection coefficients between lead specimen and output bar.

Interface	Lead → Steel	Lead → Aluminum	Lead → Nylon
Transmission coefficient	0.5552	1.075	1.8029
Reflection coefficient	−0.4448	0.075	0.8029

**Table 8:** Different material configurations for numerical SHPB test simulation.

*	Setup 1	Setup 2	Setup 3	Setup 4
Striker	Steel/20/40	Steel/20/40	Steel/12/38	Steel/12/38
Input bar	Steel/20.5/1900	Steel/20.5/1900	Steel/12/1380	Steel/12/1380
Output bar	Steel/20.5/1300	Aluminum/20.5/1300	Steel/12/1010	Aluminum/12/1010

* Material/Diameter (mm)/Length (mm).

**Table 9:** SHPB test conditions at room temperature (293 K).

T = 293 K	Test 1	Test 2	Test 3	Test 4	Test 5	Test 6
True Strain Rate (s^−1^)	1200	1750	1850	2400	2650	3800

**Table 10:** SHPB test conditions for a constant strain rate (1850 s^−1^).

ϵ = 1850 s^−1^	Test 1	Test 2	Test 3	Test 4	Test 5
Initial temperature (K)	293	323	373	423	473

**Table 11:** Material properties.

$C_{p}$ $\;(\mathrm{Jk}g^{-1}K^{-1})$	$\rho_{s}$ $\;(\mathrm{kg}.m^{3})$	β
129	10,940	0.9

**Table 12:** Perfect plasticity model parameter.

A (MPa)	n (-)	k (K)	$\dot{\varepsilon_{0}\;}$ $\left(s^{-1}\right)$
15.99	0.2285	−338.02	1850
